# An improved adaptive triangular mesh-based image warping method

**DOI:** 10.3389/fnbot.2022.1042429

**Published:** 2023-01-23

**Authors:** Wei Tang, Fangxiu Jia, Xiaoming Wang

**Affiliations:** College of Mechanical Engineering, Nanjing University of Science and Technology, Nanjing, China

**Keywords:** image stitching, mesh deformation, image alignment, color consistency, combining strategy

## Abstract

It is of vital importance to stitch the two images into a panorama in many computer vision applications of motion detection and tracking and virtual reality, panoramic photography, and virtual tours. To preserve more local details and with few artifacts in panoramas, this article presents an improved mesh-based joint optimization image stitching model. Since the uniform vertices are usually used in mesh-based warps, we consider the matched feature points and uniform points as grid vertices to strengthen constraints on deformed vertices. Simultaneously, we define an improved energy function and add a color similarity term to perform the alignment. In addition to good alignment and minimal local distortion, a regularization parameter strategy of combining our method with an as-projective-as-possible (APAP) warp is introduced. Then, controlling the proportion of each part by calculating the distance between the vertex and the nearest matched feature point to the vertex. This ensures a more natural stitching effect in non-overlapping areas. A comprehensive evaluation shows that the proposed method achieves more accurate image stitching, with significantly reduced ghosting effects in the overlapping regions and more natural results in the other areas. The comparative experiments demonstrate that the proposed method outperforms the state-of-the-art image stitching warps and achieves higher precision panorama stitching and less distortion in the overlapping. The proposed algorithm illustrates great application potential in image stitching, which can achieve higher precision panoramic image stitching.

## Introduction

Image stitching algorithm to mosaic two or more images into a panorama image to create a larger image with a wider field of view is the oldest and most widely used in computer vision (Szeliski, 2007; Nie et al., 2022; Ren et al., 2022). Earlier, the methods estimate a 2D transformation between two images focus on the global warps that include similarity, affine, and projective ones (Brown and Lowe, 2007; Chen and Chuang, 2016). Thus, the global warps are usually not flexible enough for all types of scenes like low-alignment quality images and parallax images. Furthermore, the holy grail of image stitching is to seamlessly blend overlapping images, even in scenes of distortion and parallax, to provide a panorama image that looks as natural as possible (Zaragoza et al., 2013).

While image stitching based on global warps (Zhu et al., 2001; Brown and Lowe, 2007; Kopf et al., 2007) can achieve good results, it still suffers from local distortion and is unnatural. The global warps estimate the global transformation, and they are robust but often not flexible enough. To address the model problem of global warps, many local warp models have been proposed, such as the dual-homography warping (DHW) (Gao et al., 2011), smoothly varying affine (SVA) (Lin et al., 2011) stitching, as-projective-as-possible (APAP), single-perspective warps (SPW), and so on. Unlike global warps, the above methods adopt multiple local parametric warps as the primary (Zaragoza et al., 2013; Liao and Li, 2019; Li et al., 2019; Guo et al., 2021), which is more flexible than the global warps. The DHW divides the image into two parts: a distant back plane and a ground plane, and it can seamlessly stitch most scenes. To achieve flexibility, Lin et al. (2011) proposed a smoothly varying affine stitching field that is defined over the entire coordinate frame, which is better for local deformation and alignment. Therefore, it is more tolerant of parallax than traditional global homography stitching. Instead of adopting an optimal global transformation, APAP estimates local space transformations to align every local image patch accurately.

Local parametric methods use spatially varying models to represent the motion of different image regions (Gao et al., 2011; Zaragoza et al., 2013; Chen et al., 2018). Compared to global methods, the higher degrees of freedom make them more flexible in handling motion in complex scenes but also make the model estimation more difficult (Chen et al., 2018; Liao and Li, 2019) proposed two single-perspectives warps for image stitching. The first parametric warp combines dual-feature-based APAP with quasi-homography. The second mesh-based warp is to achieve image stitching by optimizing a sparse and quadratic total energy function. Inspired by the Liu et al. (2009), many mesh-based warps (Li et al., 2015; Lin et al., 2016) have been proposed, which divide the source image into a uniform grid mesh.

In Liao and Li (2019), the stitching panorama looks as natural as possible when the source image has lots of lines; on the contrary, the stitching results represent noticeable ghosting in the curved areas and irregular object regions, such as the curve on the ground and the orange bag in the blue and red box in **Figure 6**. Meanwhile, **Figure 6** illustrates the results of APAP which looks much better than global alignment, but visible ghosting still appears in some areas, such as the orange bag in the blue box picture.

To address the above problem with distortion and ghosting in the stitched images, we improved our method's meshing and combined our warps with APAP. In this study, we propose an improved mesh-based image stitching method. To optimize the quadrilateral grid cells, we introduce an innovative triangular mesh strategy. The mesh vertices include two parts: APAP and matched feature vertices. The APAP vertices belong to uniform vertices, which can preserve the flexibility of the APAP algorithm. Thus, the matched feature vertices, which are non-uniform, can make a few artifacts in overlapping regions. We then design a color constraint term in the energy function, and the global alignment term includes two transformations for the mesh vertices. The matched feature vertices can reduce ghosting in overlapping areas in the function term. Finally, to reduce distortion in non-overlapping areas, we combine our method with APAP warp and give the weight value by calculating the distance between the vertex and the nearest matched feature point to the vertex. The comparative experiments prove that the alignment accuracy of our method is higher than the APAP warp. In summary, our three contributions are as follows:

(1) We introduce an improved mesh deformation model, including two-part vertices: non-uniform and uniform vertices. Then, the cell in our method is changed from quads to triangles, which is a novel mesh different from the conventional ways. Thus, results show that our model makes few artifacts in overlapping regions.

(2) We also design a new deformation function, which includes the data term, global alignment term, and color smoothness term. Unlike other warps, the color smoothness term can constrain the overlapping regions' smoothness.

(3) We give a new strategy of combining our method with APAP warp to obtain its flexibility.

We compare our method with the state-of-the-art image stitching methods, and the comparison experiments illustrate that our method outperforms all other methods in preserving local details and with few artifacts in overlapping regions. This syudy is organized as follows. Section 1 is the introduction. Section 2 shows the related work of image stitching. Section 3 introduces the proposed method for image stitching in detail. In Section 4, the results and comparison experiments with other algorithms were presented. Finally, Section 5 shows the conclusion of this article.

## Related work

Image stitching has been widely used in computer vision and many applications. This section will give a brief finding on image stitching.

### Multi-homography method for image stitching

A single global homography matrix can be used to express the relationship between images when the scenes are approximately in the same plane. The actual scenes are often complex with multiple planes; thus, employing the global homography to align images in the overlapping region is usually not flexible enough to provide high-precision alignment. Gao et al. (2011) proposed a dual-homography warping, which divides the image into two parts: a distant back plane and a ground plane, and it can seamlessly stitch most scenes. The method can improve alignment accuracy, but for complex scenes with multiple planes, this method incorrectly divides the different planes into one structure, which will lead to alignment errors. Hence, Yan et al. (2017) proposed a robust multi-homography image composition method. By calculating different homographies from different types of features, multiple homographies are then blended with Gaussian weights to construct a panorama. When the scene is complex, and there are multiple planes, the method based on the simple multiple homographies is ineffective for alignment. Many methods (Chen and Chuang, 2016; Medeiros et al., 2016; Zheng et al., 2019) based on planar segmentation were provided to align images. Zheng et al. (2019) proposed a novel projective-consistent plane-based image stitching method. According to the normal vector direction of the local area and the reprojection error of the aligned image, the overlapping area of the input image is divided into several projection-uniform planes.

### Image stitching based on mesh deformation

The main idea of image stitching based on mesh deformation (Liu et al., 2009; Zaragoza et al., 2013; Chen and Chuang, 2016; Chen et al., 2018; Liao and Li, 2019) is to mesh the image, transform the deformation of the image into the redrawing of the mesh, and then correspond the deformation of the mesh to the deformation of the image. This method enables the vast majority of matched feature point pairs to be completely aligned. Such methods realize image stitching by constructing an energy function for mesh vertices, and different results can be achieved by adding different constraints to the energy function. Liu et al. (2009) proposed a content-preserving warp (CPW) for video stabilization. This method divides the aligned image into multiple grid units and then constructs an energy function for the grid vertices consisting of data items, similar transformation items, and global alignment items and obtains the redrawn vertex coordinates by minimizing the energy function. The vertex coordinates of the grid where the feature points are located are optimized by the energy function, which can protect the shape of the important area of the image from being changed during the transformation. Zaragoza et al. (2013) proposed a moving direct linear transformation (Moving DLT) method to obtain the local homography matrix for each grid cell. The method added a weight value for each grid when calculating the local homography matrix. Liao and Li (2019) and Jia et al. (2021) proposed an image stitching method combining point features and line features and introduced global collinear structures into an energy function to specify and balance the desired characters for image stitching.

### Seam-driven image stitching

When the image parallax is large, the image stitching method based on spatial transformation can no longer obtain accurate results. For such image stitching problems with large parallax, the more effective method is the image stitching approach based on stitching seam (Gao et al., 2013; Zhang and Liu, 2014; Lin et al., 2016; Chen et al., 2022). Gao et al. (2013) proposed an image stitching method based on seam driven, which obtains the final homography matrix based on the quality of the stitching seam. Zhang and Liu (2014) proposed a method for local alignment using CPW near stitching seam to achieve large parallax image stitching and combined homography transformation with content-preserving warp. The experiment results illustrated that their method could stitch images with large parallax well. A superpixel-based feature grouping method (Lin et al., 2016) was proposed to optimize the generation of initial alignment hypotheses. To avoid generating only potentially biased local homography hypotheses, the hypothesis set was enriched by combining different sets of superpixels to generate additional alignment hypotheses. Then, the method evaluated the alignment quality of the stitching seam to achieve the final panorama stitching. Chen et al. (2022) proposed a novel warping model based on multi-homography and structure preserving. The homographies at different depth regions were estimated by dividing matched feature pairs into multiple layers. Collinear structures were added to the objective function to preserve salient line structures. Thus, an optimal stitching seam search method based on stitching seam quality assessment was proposed.

## Our approach

**Figure 1:**
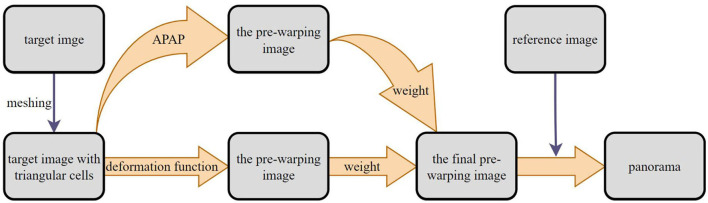
The schematic diagram of the proposed image stitching method.

This section will give a detailed presentation of our image stitching approach. We first describe the traditional global homography model to pre-align the reference and the target image; a roughly global homography is obtained to help refine image stitching in the later sections. Then, we introduce the triangular mesh deformation and give the total energy function to get the coordinates of triangular mesh vertices after deformation. Finally, a regularization parameter is introduced to balance the global and local vertices after deformation; hence, the final result can be automatically adjusted by the input images. Major steps of our proposed scheme, as shown in Figure 1.

### The similarity projective transformations

Given a pair of matching points *p* = [*x y*]^*T*^ and *p*′ = [*x*′ *y*′]^*T*^ across overlapping images *I* and *I*′. The homography model can be represented as follows


(1) $$\begin{array}{l} {\tilde{p}}^{\prime }=\text{H}\tilde{p}, \end{array}$$

Where $\tilde{p}$ is *p* in homogeneous coordinates, $\tilde{p}={\left[x\;y\;1\right]}^{T}$, and ${\tilde{p}}^{\prime }={\left[x^{\prime }\;y^{\prime }\;1\right]}^{T}$. **H** ∈ ℝ^3 × 3^ denotes the homography matrix and $\text{H}={\left[h_{1}\;h_{2}\;h_{3}\right]}^{T}$. In inhomogeneous coordinates,


(2) $$\begin{array}{l} x^{\prime }=\frac{h_{1}^{T}{\left[\begin{array}{ccc} x & y & 1 \end{array}\right]}^{T}}{h_{3}^{T}{\left[\begin{array}{ccc} x & y & 1 \end{array}\right]}^{T}}\text{ and }y^{\prime }=\frac{h_{2}^{T}{\left[\begin{array}{ccc} x & y & 1 \end{array}\right]}^{T}}{h_{3}^{T}{\left[\begin{array}{ccc} x & y & 1 \end{array}\right]}^{T}}. \end{array}$$

Taking a cross product on both sides of Equation (1), we can obtain the following:


(3) $$\begin{array}{l} 0_{1\times 3}=\left[\begin{array}{ccc} 0_{1\times 3} & -{\tilde{p}}^{T} & y^{\prime }{\tilde{p}}^{T}\\ {\tilde{p}}^{T} & 0_{1\times 3} & -x^{\prime }{\tilde{p}}^{T}\\ -y^{\prime }{\tilde{p}}^{T} & x^{\prime }{\tilde{p}}^{T} & 0_{1\times 3} \end{array}\right]\left[\begin{array}{c} h_{1}\\ h_{2}\\ h_{3} \end{array}\right]. \end{array}$$

There only two rows of the 3 × 9 matrix in Equat9ion (3) are linearly independent, and we let $a_{i}\in {\mathbb{R}}^{2\times 9}$ be the first-two rows of Equation (3) computed for the i-th datum for a set of N matched points ${\left\{p_{i}\right\}}_{i=1}^{N}\text{and}{\left\{p_{i}^{\prime }\right\}}_{i=1}^{N}$, we can obtain *h* by the following


(4) $$\begin{array}{l} \hat{\text{h}}=\arg\underset{\text{h}}{\min}\sum ||a_{i}\text{h}{||}^{2}=\arg\underset{\text{h}}{\min}||\text{A}\text{h}{||}^{2}. \end{array}$$

With the constraint ||h|| = 1, where matrix $\text{A}={\left[a_{1}\;a_{2}\ldots \;a_{i}\right]}^{T}$. Given the estimated **H** (reshaped from $\hat{\text{h}}$), to align the images, the arbitrary pixel in the source image *I* is warped to the target image *I*′ by Equation (1). Thus, the details can be found in Lin et al. (2015).

### Triangular mesh deformation

The image stitching based on mesh deformation usually uses the quadrilateral grid, but the warp could still have less distortion at the position of the matched feature points. Therefore, we propose a triangular mesh cell, including APAP and matched feature vertices.

#### Mathematical setup

Inspire by the work of Li et al. (2019), they introduced the planar and spherical triangulation strategies and approximated the scene as a combination of adjacent triangular facets. This inspired us, so we partitioned the source image into a triangular mesh of a series of cells and took the matching points and APAP's vertices as our triangular mesh vertices. Then, a triangulation-based local alignment algorithm for image stitching is proposed, which could compensate for the weaknesses of the quadrilateral grid deformation.

For ease of explanation, we take the two image stitching pair as an example and let *I*′, *I*, and Î to denote the reference image, the target image, and the final warping image. We keep the reference image *I*′ fixed and warp the target image *I*. Thus, the vertices in the image *I*, *I*′, and Î are denoted as *V*, *V*′, and $\hat{V}$.

**Figure 2:**
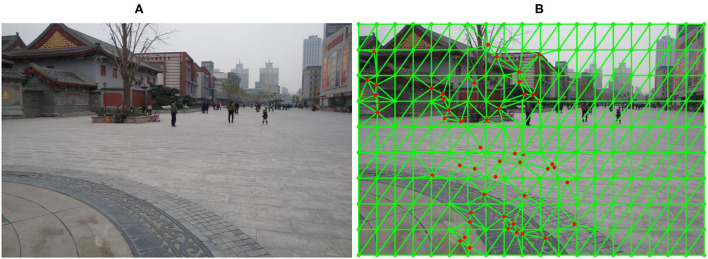
View triangulation results on the target image. **(A)** The template image and **(B)** the triangular mesh image. The green dots are APAP vertices, and the red dots denote matched feature vertices.

Unlike traditional quadrilateral grid deformation warps, we partition the source target image *I* into a series of triangular cells by Delaunay triangulation (Edelsbrunner et al., 1990). For each cell, three vertices are more stable than the four vertices in the quadrilateral cell. To make the image stitching warp more stable, we choose a series of APAP's vertices as the triangular cell vertices and add n-matched feature points as vertices into the original vertices. Therefore, the target image is partitioned into many cells, including two parts: APAP and matched feature vertices. Figure 2 illustrates a warp learned with 250 vertices cells for an image pair.

In addition, after building mesh grids for the target image *I*, where *V*_*i, j*_ is the grid vertex at position (*i, j*). The target image is composed of many cells which have three vertices, and we index the grid vertices from 1 up to n; we reshape all vertices into a 2n-dimension vector $V={\left[x_{1}\;y_{1}\;\ldots \;x_{n}\;y_{n}\right]}^{T}$; then, the mesh deformation vertices which correspond to the target image vertices are formed into $\hat{V}={\left[{\hat{x}}_{1}\;{ŷ}_{1}\;\cdots \;{\hat{x}}_{n}\;{ŷ}_{n}\right]}^{T}$. Each cell has four vertices in Liao and Li (2019), so different from Liao and Li (2019), the mesh deformation cell has three vertices in our approach.

In Liao and Li (2019), each feature point *p* can be characterized as a bilinear interpolation of its four enclosing grid vertices. Thus, similar to Liao and Li (2019), for any feature point *p* in the triangular cell, which can be expressed as a linear interpolation of the triangular vertices v1, v2, and v3. Different from the bilinear interpolation, barycentric coordinate system (Koecher and Krieg, 2007) can denote any point which is inside the triangle cell well. So, the feature point *p* can be characterized as follows:


(5) $$\begin{array}{l} \varphi \left(p\right)=w_{1}v_{1}+w_{2}v_{2}+w_{3}v_{3}, \end{array}$$

Where *w*_1_, *w*_2_, and *w*_3_ denote the weight of each vertex, respectively, the higher the weight, the closer the point is to the vertex, and *w*_1_+*w*_2_+*w*_3_ = 1. If we get a known point inside the triangle, the weights will be obtained by solving a binary system of linear equations.

Assuming that the weights are fixed, thus the corresponding point *p*′ that is after mesh deformation can also be characterized as $\varphi \left(\hat{p}\right)=w_{1}{\hat{v}}_{1}+w_{2}{\hat{v}}_{2}+w_{3}{\hat{v}}_{3}$. Subsequently, any constraint on the point correspondences, which are inside the triangle can be expressed as a constraint on the three vertex correspondences.

#### Energy function definition

Inspired by the study of the content-preserving warps Liu et al. (2009), we construct the total energy function *E* that includes the following three parts: data term, global alignment term, and color smoothness term.


(6) $$\begin{array}{l} E\left(\hat{V}\right)=E_{D}\left(\hat{V}\right)+\omega_{G}E_{G}\left(\hat{V}\right)+E_{C}\left(\hat{V}\right), \end{array}$$

**Figure 3:**
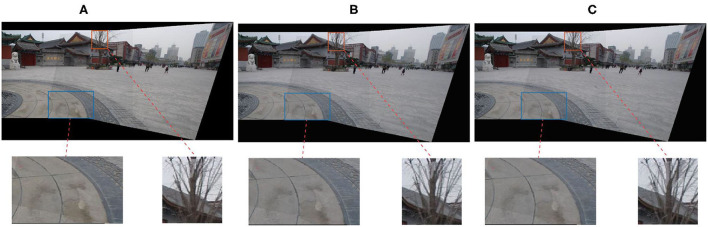
Comparison of stitching results with different ω_*G*_. **(A)** ω_*G*_ = 0, **(B)** ω_*G*_ = 10, and **(C)** ω_*G*_ = 5000.

Where *E*_*D*_ denotes the data term that addresses the alignment issue by enhancing the feature point correspondences, *E*_*G*_ is the global alignment term, and *E*_*C*_ addresses a color smoothness issue by protecting the vertices' intensity and its neighboring region. The deformed vertex $\hat{V}$ can be calculated by the above formula, then mapping the deformation of the mesh to the deformation of the image to obtain the final panorama. The above minimization problem is easily solved using a standard spares linear solver. We use texture mapping to extract the final image when we get the deformed vertices. The weight ω_*G*_ = 10 in our implementation. Figure 3 shows the stitching results of different ω_*G*_. Theoretically, the larger ω_*G*_ is, the better the alignment at the matched feature vertex positions of the stitching results; the blue box in Figure 3 verifies this point. Thus, ω_*G*_ is too large, which means the weight of the global alignment term is too large. As shown in the red box in Figure 3, too much weight of data items will affect the stitching effect of other regions.

## A. Data term

The data term *E*_*D*_ is defined the same way as Liu et al. (2009). Thus, the feature point *p* which is in the mesh cell can be denoted by the triangular vertices of its enclosing grid cell. To align *p* to its matched location *p*′ after deformation, we define the data term as follows:


(7) $$\begin{array}{l} E_{D}=\underset{i}{\sum }\|\overset{3}{\underset{i=1}{\sum }}w_{i,k}{\hat{V}}_{i,k}-p_{i}^{\prime }{\|}^{2} \end{array}$$

Where $\hat{V}$ is the unknown coordinate of mesh vertices to be estimated, ω_*i, k*_ is the interpolation coefficient, which is obtained by the mesh cell, that contains *p*_*i*_ in the target image (Equation 5), and $p_{i}^{\prime }$ is the corresponding feature point in the reference image.

## B. Global alignment term

To align the grid vertices and avoid unnecessary moving of the vertices from their pre-warped positions, we construct an improved global term to provide a good estimation. We redefine the global term *E*_*G*_ as the summation in the L2 norm of the difference between the origin vertex and its deformation.


(8) $$\begin{array}{l} E_{G}=\underset{j}{\sum^{\text{​}}}{\left\|V^{*}{\hat{V}}_{j}-{\left(V^{*}\right)}^{2}\right\|}^{2} \end{array}$$

(9) $$\begin{array}{l} V_{j}^{*}=\{\begin{array}{cc} p_{j}^{\prime }, & if\;V_{j}\text{ is feature point vertex}\\ H_{APAP}V_{j}, & \text{other vertices} \end{array}, \end{array}$$

Where $p_{i}^{\prime }$ denotes the matching feature point in the reference image *I*′, *H*_*apap*_ is the local homography in Zaragoza et al. (2013) and *j* is the cell vertices index. *V* and $\hat{V}$ are the corresponding vertex in the target image triangular cell and its deformation.

## C. Color similarity term

To constrain the smoothness of color models with a connected neighboring region and let these selected intensities remain close after the mesh deformation, we design this color similarity term. Assuming that the overlapping image region with any points has the same intensities. Thus, we can obtain the intensity difference value between the two overlapping image parts.


(10) $$\begin{array}{l} E_{c}=\sum_{\Omega }\sum_{(x,y)=Q}{\left\|{\hat{I}}_{\Omega }(\hat{x},\hat{y})-I_{\Omega }^{\prime }\left(x^{\prime },y^{\prime }\right)\right\|}^{2} \end{array}$$

Where *Q* denotes the feature point set, which is in the overlapping image region. Here, Ω denotes the point connected neighboring area at position $\left(\hat{x},ŷ\right)$ and its corresponding (*x*′, *y*′). Ω is set to 9 × 9 in our experiment.

### Joint optimization

After we obtain a warped version of this triangular mesh vertices by the above energy function. The overlapping image area in the target image and reference image can stitch well, and the mosaic image has a good performance. The feature points have a good match pair only on the overlapping region, and if we only get the warped version by the energy function, the stitching result may have an unnatural visual effect on the non-overlapping area. Hence, we update the final warped vertices by controlling the relative amount of the vertices obtained with APAP warps injected into the vertices obtained by the energy function way in a soft manner, which can be auto-adjusted further by the origin image pair. The final vertices can be denoted as follows:


(11) $$\begin{array}{l} {\tilde{V}}_{i}=c_{i}^{1}{\hat{V}}_{i}+c_{i}^{2}{\bar{V}}_{i}, \end{array}$$

Where, ${\tilde{V}}_{i}$ is the final triangular cell vertex after deformation, *V*_*i*_ is the cell vertex in the target image *I*, ${\bar{V}}_{i}=H_{apap}V_{i}$, and ${\hat{V}}_{i}$ denotes the vertices after deformation by the energy function. *H*_*apap*_ can find the details in Zaragoza et al. (2013), APAP computes a local homography for each image patch for high-precision local alignment, so we use each homography in this study. *c*^1^ and *c*^2^ are weighting coefficients. We also make *c*^1^+*c*^2^ = 1, and *c*^1^ and *c*^2^ are between 0 and 1. They are identified by the following equations:


(12) $$\begin{array}{l} c_{i}^{2}=\frac{\min\left(\frac{D_{i}}{\max\left(D_{i}\right)},\gamma \right)}{\gamma },c_{i}^{1}=1-c_{i}^{2} \end{array}$$

(13) $$\begin{array}{l} D_{i}=\min\left(d_{i}\left(k\right)\right),k=1,2,3\ldots \end{array}$$

(14) $$\begin{array}{l} d_{i}\left(k\right)=Dist\left(V_{i},P\left(k\right)\right), \end{array}$$

**Figure 4:**
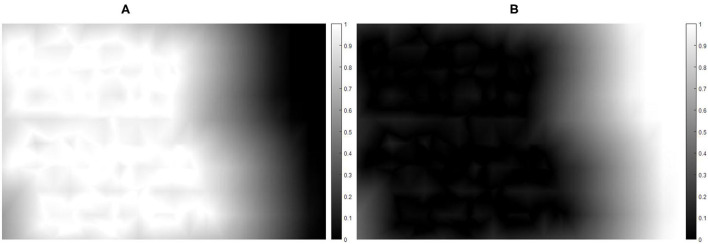
Weight map of the target image. **(A)** Weight map of content-preserving warps and **(B)** weight map of APAP warps. The color denotes the weight value, which is between 0 and 1.

**Figure 5:**
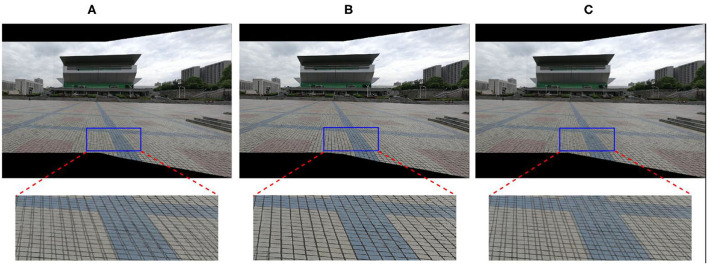
Comparisons with APAP and global homography. **(A)** APAP, **(B)** our method, and **(C)** global homography.

Where Dist(·) represents the function to calculate the distance between two points, *P* is the feature point sequence of *p*_1_, *p*_2_,…, γ is an adjustable parameter, in fact, as γ → 1 the shortest distance when the weight is equal to 1 between vertex and the matched feature points is the largest. Thus, *V*_*i*_ is the location of the i-th location in the image cell vertices. As shown in Figure 4, when the vertex is near the over from the matched feature points regions (the overlapping regions), the content-preserving warps have a high weight to ensure accurate alignment. On the contrary, the APAP warps have a high weight for fewer distortions for vertices far from the overlapping regions. Therefore, the final warp has good performance by using the weight combination. Figure 5 shows the comparison results with APAP and global homography.

## Experiments

To verify the effectiveness of the proposed image stitching method, we test the method by subjective and objective assessments on pairwise datasets. In this section, we illustrate several representative image pair stitching results for comparing our warp for image stitching with several state-of-the-art stitching methods. First, we show a quantitative evaluation of the alignment accuracy for comparing our method against the state-of-the-art image stitching methods, namely, APAP, global homography, APAP, AutoStich, and SPW. Second, we give a quantitative evaluation of pairwise alignment by our image stitching way and several state-of-the-art methods. The mesh-based warps have a good performance; therefore, we ran a series of tests. Thus, the experimental parameters of the comparative paper are also consistent with the original paper.

In our experiment, we use VLFeat (Vedaldi and Fulkerson, 2010) library to extract and match SIFT (Lowe, 2004) feature key points and run RANSAC to remove mismatches and match feature points by Jia et al. (2016). Codes are implemented in MATLAB (some codes are in C++ for efficiency) and run on a desktop PC with Intel i3-10100 3.6 GHz CPU and 16GB RAM. Then, all the image pairs in our test are contributed by the authors of Li et al. (2017). For parameter settings, γ = 0.8, the number of the APAP vertex is set to 5 × 6, and the matched feature vertex is set to 0.7x the total number of the matched feature points. As shown in Figure 3, if ω_*G*_ is too small then the vertices distortion becomes serious, and if ω_*G*_ is too large, then the region outside the vertex is severely distorted. Thus, ω_*G*_ is set to 10 in the experiment. The experimental parameters of the comparison algorithm are consistent with its original paper.

### Qualitative evaluation of pairwise stitching

**Figure 6:**
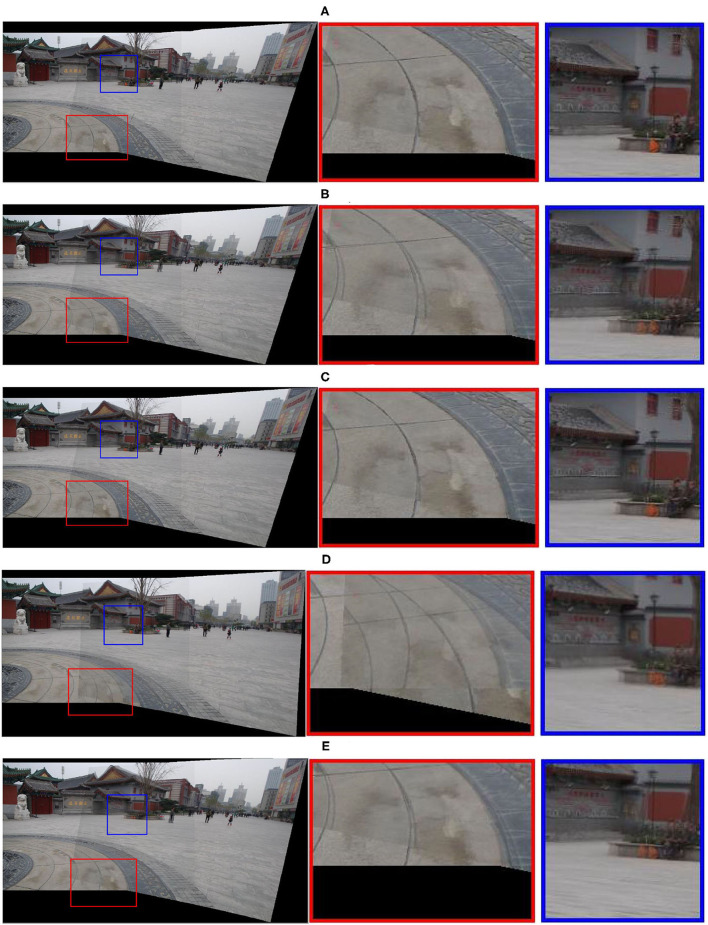
Comparisons with state-of-the-art image stitching techniques on the Temp image dataset. From top to bottom, each row is **(A)** our method, **(B)** global homography, **(C)** APAP, **(D)** AutoStich, and **(E)** SPW. The red boxes and blue boxes show the stitching details clearly stated.

Figure 6 depicts the result of image stitching on the Temp image pair. Each row illustrates a panorama result of different methods, and the green and blue box regions are enlarged for a wide view of the local details. As we can see, all the results have a good performance. Nevertheless, our method has a better performance on the details. The global homography and AutoStitch could not align two images well using a global 2D transformation, in addition to the stitching results suffering from ghosting, such as the curves on the ground in the green rectangle and the orange bag being duplicated in the blue zoomed-in rectangle. Considering the limitations of global transformation, the APAP method shows a fine stitching result as shown in Figure 6C; however, the details in the APAP results are not good as our method, comparing the white arched logo in Figures 6A, C, it can be seen that our result has few artifacts. As shown in Figures 6A, B, D, the orange bag in the blue zoomed-in rectangle has few artifacts in our results. The SPW method has a weakness in the image with few lines, the detail is illustrated in Figure 6E, and there is obvious misalignment. Contrast the above methods with our method, which has less “ghostly” with few artifacts. Especially, the curves on the ground, the white arched logo on the wall, and the orange bag in the blue zoomed-in rectangle have few artifacts, as shown in the first row of Figure 6A, so our method has the best stitching quality. The better performance is due to our approach adding a tight constraint into the mesh warps and combining our method with the APAP warp.

**Figure 7:**
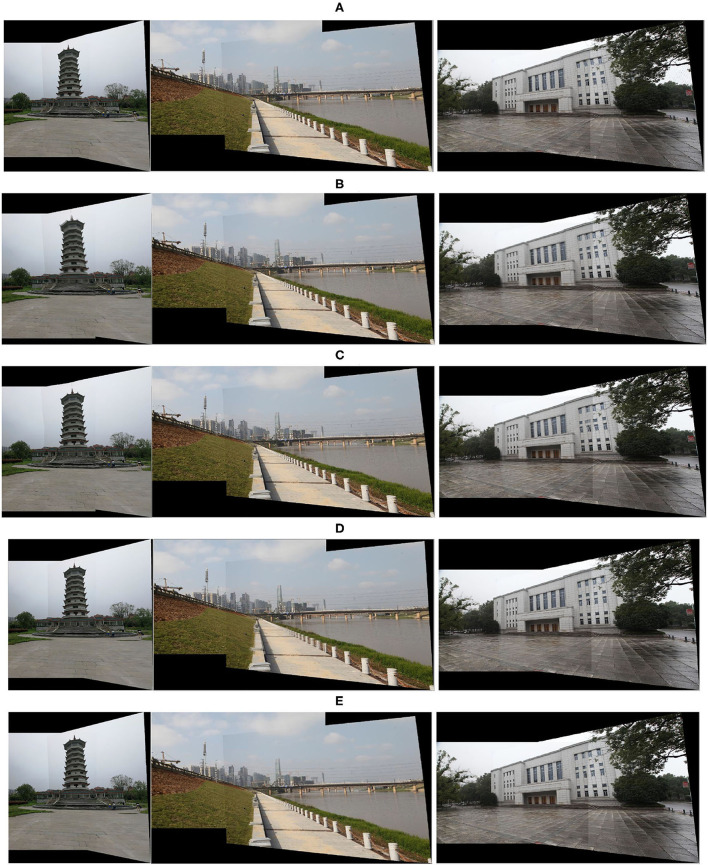
Comparison results for different scenes. From top to bottom, the image stitching results are **(A)** our method, **(B)** global homography, **(C)** APAP, **(D)** AutoStitch, and **(E)** SPW, respectively. Here, from left to right, the scenes are the *tower, riverbank*, and *theater*.

To comprehensively demonstrate the effectiveness of our image stitching method, we compare the final stitching results on a different scene. As shown in Figure 7, from left to right, the stitching results are the tower, riverbank, and theater, respectively. In the results of the riverbank, the round pillar misalignments are shown in the AutoStitch method. The other stitching method has a good performance on the riverbank. However, our method shows the *roads, wires*, and *buildings* on the riverbank more clearly. As shown in *tower*, the global homography method shows an obvious “ghostly,” and the gaps in the paving exhibit non-uniform distortions over the image. In the SPW result, the top of the tower is duplicated. Thus, all of the results introduce obvious distortion or ghosting, as indicated in Figure 7. As for scene *theater*, the gaps in the paving show less ghosting than the other methods because the authors of SPW combine point and line features in the mesh-based warp. Then, the building on the overlapping region exhibited more ghosting than our method. Generally speaking, our method shows less distortion and ghosting results.

### Quantitative evaluation of alignment

To quantify the alignment accuracy of our proposed method, we calculate the structural similarity index (SSIM) (Wang et al., 2004) along the overlapping region points as an evaluation standard. The SSIM is usually used to describe the alignment accuracy on the different images. The quantitative results are shown in Table 1, which includes five methods tested data from seven scenes. As shown in Table 1, our method yields the highest similarity value in five scenes, and our method is next to the highest value in the other two scenes. Our average similarity value is 0.9426, 1.5% higher than SPW, 5.5% higher than AutoStitch, 3.3% higher than global homography, and 1.8% higher than APAP. A comprehensive visual comparison is demonstrated in Figures 6, 7. Our method performs better than all the other methods in preserving local details and being artifact-free in overlapping regions.

**Table 1:** Comparison of the SSIM of different scenes (the global homography is abbreviated as GH).

	**Railtracks**	**Temp**	**Tower**	**Theater**	**Riverbank**	**Racetracks**	**Worktable**	**Average**
Our	**0.936**	**0.945**	**0.963**	**0.947**	0.959	**0.898**	0.949	**0.943**
GH	0.884	0.905	0.945	0.896	0.949	0.867	0.936	0.913
APAP	0.909	0.939	0.912	0.918	**0.965**	0.887	**0.953**	0.926
AutoStitch	0.898	0.913	0.946	0.921	0.959	0.864	0.751	0.893
SPW	0.922	0.911	0.946	0.933	0.960	0.880	0.947	0.928

The best value is shown in bold.

## Conclusion

We have proposed an improved adaptive triangular mesh-based image stitching method. First, without sacrificing the accuracy of alignment, a non-uniform triangular mesh is set over the image to improve alignment accuracy. The non-uniform grid includes uniform and non-uniform vertices, and the non-uniform vertices are from the matched feature points, which provide good constraints on overlapping areas and is a novel method. Second, an improved deformation function is constructed to obtain deformed vertices. To constrain the smoothness of the color model, we introduced a color similarity term in the deformation function. Finally, we give a novel strategy for combining our method with APAP warp to obtain its flexibility. The combining strategy not only absorbs the advantages of the good alignment of APAP but also can adaptively adjust its weight value. The proposed algorithm is proved on different images and compared with other methods. The experimental results illustrate that the image stitching method in this study can achieve more accurate panoramic stitching and less overlapping distortion and improve the accuracy of panoramic image stitching. The proposed method has an improvement in accuracy compared to the other methods. The mean SSIM of the proposed method is 0.9426, which is 1.5% higher than SPW, 5.5% higher than AutoStitch, 3.3% higher than global homography, and 1.8% higher than APAP. For further work, we expect to apply this method to large parallax image stitching and image stitching with moving targets.

## Data availability statement

The original contributions presented in the study are included in the article/supplementary material, further inquiries can be directed to the corresponding author.

## Author contributions

WT created the improved model and the provided initial idea, conducted the experiments, and wrote the article. FJ and XW put forward some effective suggestions for improving the structure of the article. All authors contributed to the article and approved the submitted version.
